# Soluble IL-2Rα (sCD25) Exacerbates Autoimmunity and Enhances the Development of Th17 Responses in Mice

**DOI:** 10.1371/journal.pone.0047748

**Published:** 2012-10-15

**Authors:** Shane E. Russell, Anne C. Moore, Padraic G. Fallon, Patrick T. Walsh

**Affiliations:** 1 Department of Clinical Medicine, School of Medicine, Trinity College Dublin, Dublin, Ireland; 2 National Children's Research Centre, Our Lady's Children's Hospital, Crumlin, Dublin, Ireland; 3 School of Pharmacy, University College Cork, Cork, Ireland; University of Michigan Medical School, United States of America

## Abstract

A strong association exists between mutations at the *IL2 receptor alpha chain (CD25)* gene locus and susceptibility to a number of T cell driven autoimmune diseases. Interestingly, the presence of certain *CD25* susceptibility alleles has been correlated with significantly increased levels of the soluble form of CD25 (sCD25) in the serum of patients. However, the functional consequences, if any, of this observation are unknown. We have demonstrated that elevated levels of sCD25 *in vivo* resulted in exacerbated experimental autoimmune encephalomyelitis (EAE) and enhanced antigen-specific Th17 responses in the periphery. sCD25 exerted its effects early during the Th17 developmental programme *in vitro*, through inhibiting signalling downstream of the IL-2R. Although, sCD25 did not interact with the T cell surface, it specifically bound to secreted IL-2 demonstrating its ability to act as a decoy receptor for IL-2 in the T cell microenvironment. These data identify the ability of sCD25 to promote autoimmune disease pathogenesis and enhance Th17 responses through its ability to sequester local IL-2.

## Introduction

The primary non-redundant function of interleukin-2 (IL-2) is as a mediator of peripheral T cell tolerance [1]. As well as the maintenance of peripheral tolerance, IL-2 has also been somewhat paradoxically described play an important role in driving the proliferative response of activated T cells and as a critical factor in the generation of an appropriate memory T cell response [2]. IL-2 exerts these pleiotropic effects through interacting with the heterotrimeric IL-2 receptor (IL-2R) complex comprised of α (CD25), β (CD122) and common γ (CD132) chains expressed on the surface of activated T cells [3]. The critical role for IL-2 in the maintenance of T cell tolerance is evident from studies on transgenic mice deficient for either the cytokine itself or its receptor [4], [5]. These mice develop profound autoimmunity characterized by an uncontrolled expansion of auto-reactive T cells. Further analysis has revealed the mechanistic basis for these observations, and uncovered the critical role of IL-2R signalling in maintaining the competitive fitness of peripheral regulatory T cells (Tregs) [6], and in the direct inhibition of Th17 cell responses [7]–[9]. These observations are further underscored by the strong genetic linkage between mutations at both the *IL-2* and *IL2RA/CD25* gene loci and several T cell mediated autoimmune diseases in humans [10]. However, precisely how such polymorphisms confer susceptibility to autoimmunity remains incompletely understood.

A number of specific *CD25* alleles associated with autoimmune susceptibility occur in association with enhanced levels of the soluble form of the IL-2R alpha chain (sCD25) in the serum of patients [10], [11]. However, the functional consequences of these observations are unknown. Numerous examples of soluble cytokine receptors have been described to exert immunomodulatory effects *in vivo*. These range from antagonistic (IL-1RII) or agonistic (IL-6R) effects on receptor signalling, to acting as ligand chaperones or carrier proteins (IL-4R) [12]. Although there have been several descriptions of sCD25 acting as an inhibitor of IL-2 induced T cell responses *in vitro* [10], [13], whether it plays a similar role *in vivo* has not been determined. sCD25 is known to be generated as a result of proteolytic cleavage, largely from the surface of activated T cells and levels of CD25 ‘shedding’ are directly related to the rate of proliferation of activated T cells [14]. The levels of systemic sCD25 in the steady state are known to be remarkably stable and as a consequence sCD25 has been used extensively as a biomarker reflecting inflammatory diseases and tumours characterized by T cell expansion [14]. However, whether these increased levels of sCD25 play any direct role in modulating disease has not been fully investigated.

In this study we demonstrate for the first time that sCD25 exacerbates experimental autoimmune encephalomyelitis (EAE). These effects are associated with the enhanced generation of Th17 type responses in the periphery and increased infiltration of both CD4+ Th1 and Th17 cell subsets into the central nervous system (CNS). Similar to monoclonal antibody mediated IL-2 neutralization, sCD25 also enhances Th17 responses *in vitro* and acts early during the Th17 developmental programme by inhibiting signalling downstream of the IL-2R through its ability to sequester local IL-2. These data identify a previously unappreciated role for sCD25 in the pathogenesis of autoimmune disease.

## Materials and Methods

### Mice

Female C57BL/6J mice (Charles River, Ireland) and IL-17AeGFP mice, on a C57BL/6 background, (Biocytogen, Worcester, MA, USA) aged between 6–8 weeks were utilised in experiments. Mice were housed under SPF conditions at the Institute for Molecular Medicine, St. James Hospital Dublin. All animal experiments were performed in compliance with Irish Department of Health regulations (license number B100/4272) and approved by the institutional ethical review board.

### Materials

ELISA kits for mouse IL-17A, IFNγ, IL-2 and IL-22 were purchased from ebioscience (Hatfield UK). ELISA kit for sCD25 was purchased from R&D systems (Abingdon, UK) Recombinant murine sCD25His was purchased from R&D systems. Endotoxin levels in sCD25 were determined by LAL assay and found to be lower than 0.05 EU/µg of protein. These levels were found to exert no detectable levels of immune stimulation on primary macrophages *in vitro*. All antibodies used in this study from ebioscience unless otherwise stated. Recombinant mouse IL-6 was purchased from Becton Dickinson (Oxford, UK), IL-12p70 and TGFβ from ebioscience. Anti-IL-2 neutralizing antibody (JES6-1A12) was purchased from ebioscience.

### Flow Cytometry

Cells were analysed for surface and intracellular protein expression using an LSR/Fortessa (BD). For intracellular staining, cells were stimulated with PMA 10 ng/ml and ionomycin 1 µg/ml for 6 h in the presence of Brefeldin A (Sigma) 5 µg/ml for the final 4 hrs. Fixation and permeabilisation was performed using FIX/PERM Kit (Dako) according to manufacturer's instructions. Intracellular staining was performed for IL-17A (17B7), IL-17F (eBio18F10), IFN-γ (XMG 1.2) and FoxP3 (FJK-16s) and RORγT (AFKJS-9) (ebioscience, UK). pSTAT5 (pY694) and pSTAT3 (pY705) staining was performed with Phosflow kit according to the manufacturer's instructions (Becton Dickinson, UK). Surface staining was performed for CD4 and CCR6. Cell surface staining for sCD25 was performed using anti-HIS (GG11-8F3.5.1) (Miltenyi Biotec).

### EAE Induction and sCD25 treatment

EAE was induced according to manufacturer's instructions using active EAE induction kit EK-0113 (Hooke Labs MA. U.S.A). Mice were monitored daily for signs of disease with disease severity recorded as follows 0. Normal, 1. Limp tail, 2. Wobbly gait, 3. Severe hind limb weakness 4. Complete hind limb paralysis 5. Moribund or death. Recombinant sCD25 was administered immediately prior to immunization and every 12 hours thereafter for the first 3 days. Control mice were treated with PBS.

### T cell isolation and differentiation

Naive CD4+CD62L+T cells from spleens of 8 week old mice were purified by magnetic bead separation (Miltenyi Biotec). Cells were activated with plate bound anti-CD3 (145-2C11) and anti-CD28 (37.51) both 5 µg/ml. For Th17 differentiation cells were cultured in the presence of TGF-β 5ng/ml, IL-6 10 ng/ml, anti-IFN-γ 10 µg/ml (XMG 1.2) and anti-IL-4 10 µg/ml (11B11). For Th1 differentiation cells were cultured with IL-12 10 ng/ml and anti-IL-4 10 µg/ml. iTreg cells were induced in the presence of TGF-β 5 ng/ml and rIL-2 (10 U/ml). After 72–96 hours supernatants were analysed by ELISA and cells were examined for intracellular cytokine/transcription factor expression by flow cytometry.

## Results

### sCD25 leads to exacerbated autoimmune disease and increased antigen-specific peripheral Th17 responses

**Figure 1:**
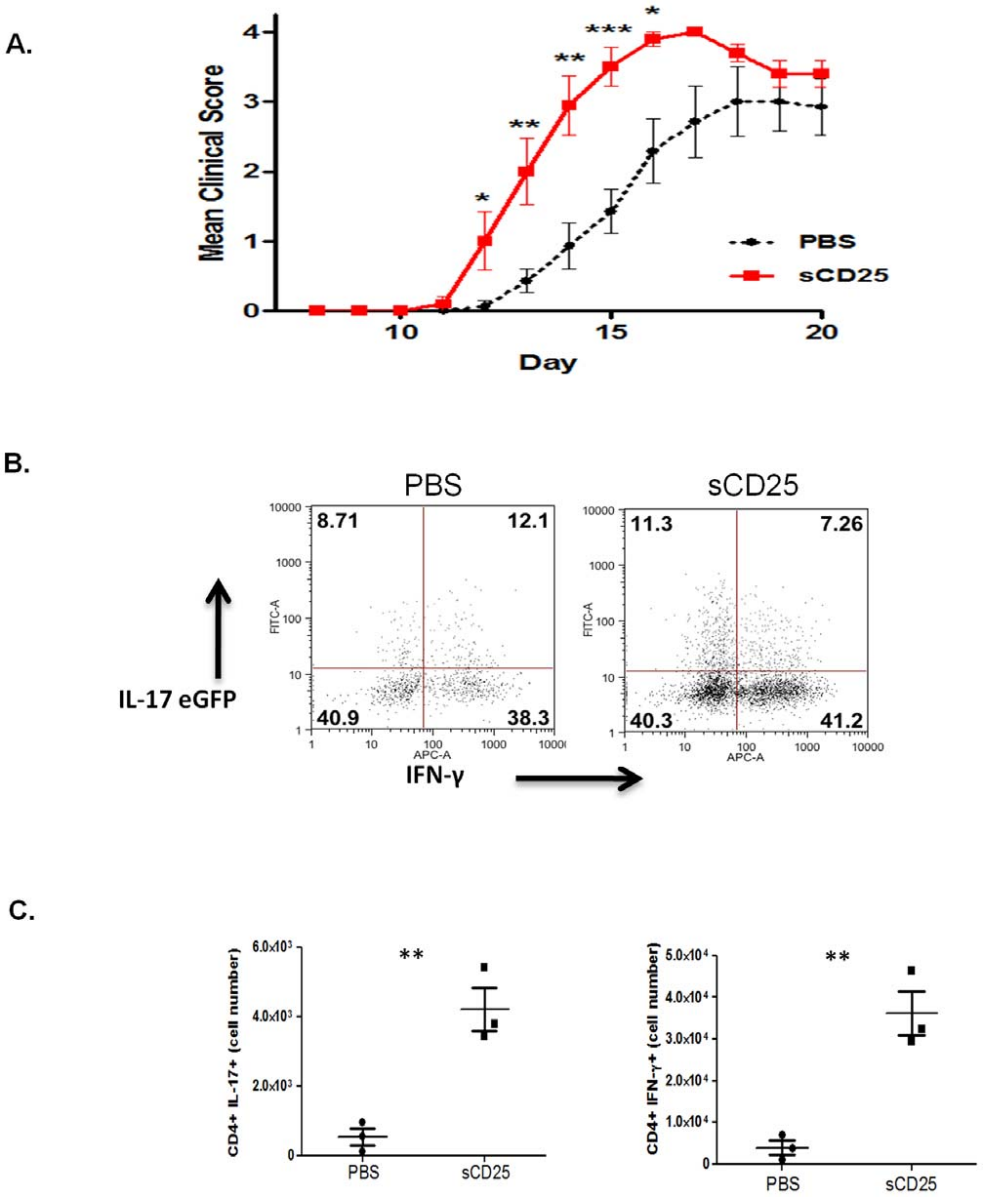
Exogenous sCD25 exacerbates autoimmunity. (**A**) MOG_33−55_ immunized C57BL/6 mice developed clinical symptoms of EAE from day 12 after immunization with a peak of disease severity observed from day 19. Subcutaneous administration of recombinant sCD25 (25 µg/mouse) immediately prior to immunization and every 12 hours thereafter for 72 hrs resulted in a significant exacerbation in severity of symptoms during disease onset and induction. 6–7 mice used per group. (**B**) Mononuclear cells harvested from spinal cords of control (PBS) and sCD25 treated IL-17A-eGFP reporter mice (3 per group) on day 15 after immunization and analysed for expression of IL-17 and IFN-γ by CD4+ cells. (**C**) Cell numbers of CD4+IL-17+ and CD4+ IFNγ+ cells in spinal cords of IL-17A-eGFP reporter mice at day 15. Data representative of mean +/− std dev of 3 mice per group and 2 independent experiments.

**Figure 2:**
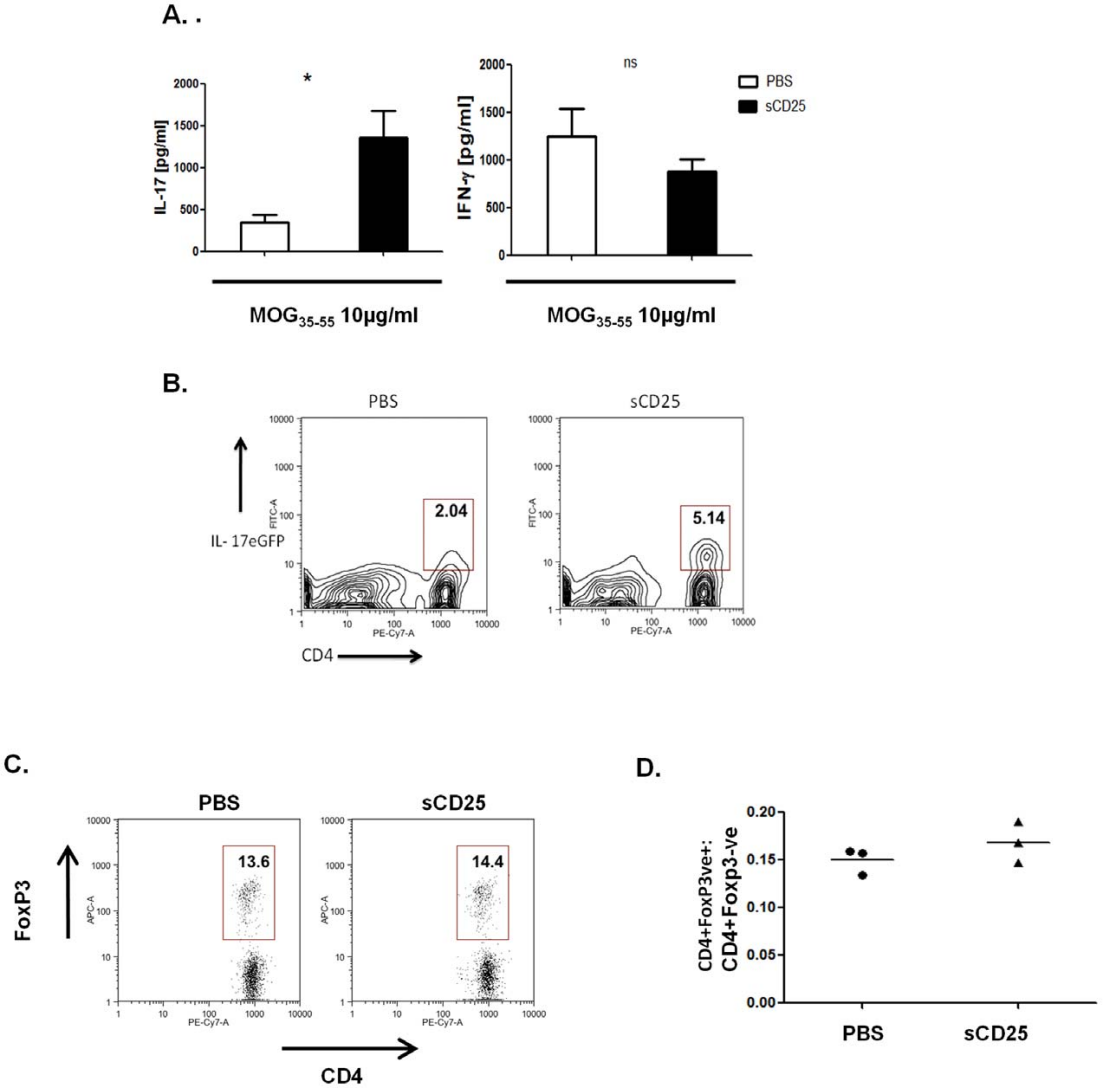
sCD25 enhances peripheral antigen specific Th17 responses. (**A**) Levels of IL-17A and IFNγ secreted by draining lymph node cells of mice immunized and treated as above (3 per group), isolated after 9 days, and restimulated *ex vivo* with MOG_33−55_ (10 µg/ml) for 72 hours. (**B**) Percentage of CD4+ IL-17A-eGFP+ve cells after treatment as above and *ex vivo* restimulation for 72 hours. (**C**) Percentage and (**D**) relative number ratios of CD4+FoxP3+:CD4+FoxP3- T cells in the draining lymph nodes of both sCD25 and control treated immunized mice. 3 mice per group were analysed. Data in D & G is representative of mean +/− standard deviation. Statistical Significance determined by unpaired student's t-test, * p≤0.05, **p≤0.01.

Specific alleles at the *CD25* gene locus, known to be associated with susceptibility to autoimmune diseases such as Multiple Sclerosis (MS), lead to increased levels of soluble CD25 in patient's serum [10]. Although such observations implicate sCD25 as having an important mechanistic role in disease pathogenesis, it is not clear how sCD25 may contribute to a loss of self tolerance. To determine what role, if any, sCD25 may play in autoimmunity we induced experimental autoimmune encephalomyelitis (EAE), a mouse model of MS, in the presence of exogenous recombinant sCD25 administered immediately prior to, and during the first 3 days after immunization. Increased levels of sCD25 during the early stages of antigen-specific T cell priming led to a significant exacerbation of disease symptoms during the onset and induction phase of the disease from day 10 through to the peak of disease after 18 days (P>0.01) (Fig. 1A). To examine the cells infiltrating into the CNS during EAE, IL-17-eGFP reporter mice were immunized with MOG, in the presence or absence of sCD25, and the expression of IL-17A or IFNγ was assessed at 15 days after induction of EAE. Although the relative percentages of infiltrating Th1 versus Th17 type cells was not altered (Fig. 1B), administration of sCD25 resulted in significantly increased numbers of both subsets in the spinal cords of treated mice at day 15 during disease induction (Fig. 1C). We also examined the effects of sCD25 administration on the generation of peripheral antigen specific T cell responses *in vivo*. Significantly, increased levels of sCD25 were found to result in increased antigen-specific T cell expression of IL-17A upon MOG antigen restimulation *ex vivo* 7 days after immunization (p>0.05) (Fig. 2A &B). Expression of IFNγ was not significantly affected (Fig. 2A). Furthermore, administration of sCD25 did not affect the levels or relative numbers of CD4+Foxp3+ regulatory T cells in immunized mice after 7 days, indicating that increased severity of EAE did not occur in association with any effects on Treg homeostasis (Fig. 2C&D). Together these data demonstrate that increased levels of sCD25 *in vivo* led to increased severity of EAE that occurs in association with enhanced generation of antigen specific Th17 responses in the periphery and increased numbers of both of CD4+ Th1 and Th17 cell subsets in the CNS. These observations are consistent with previous reports which demonstrate that administration of an anti-IL-2 neutralizing antibody leads to the spontaneous development of EAE-like symptoms in mice and also that treatment with recombinant IL-2 during the early stages of disease can offer significant protection from EAE [15]–[16].

### sCD25 enhances the development of Th17 cell responses

Conflicting studies have demonstrated both antagonistic and agonistic roles for sCD25 in the context of IL-2R signalling indicating that sCD25 could either promote or inhibit Treg responses and inhibit IL-2 mediated activation induced cell death *in vitro* [10] [17]. As sCD25 enhances the generation of peripheral autoimmune antigen-specific Th17 responses *in vivo*, we sought to determine how sCD25 might regulate these events by investigating the effects of sCD25 on the generation of Th17, Th1 and Treg responses *in vitro*.

**Figure 3:**
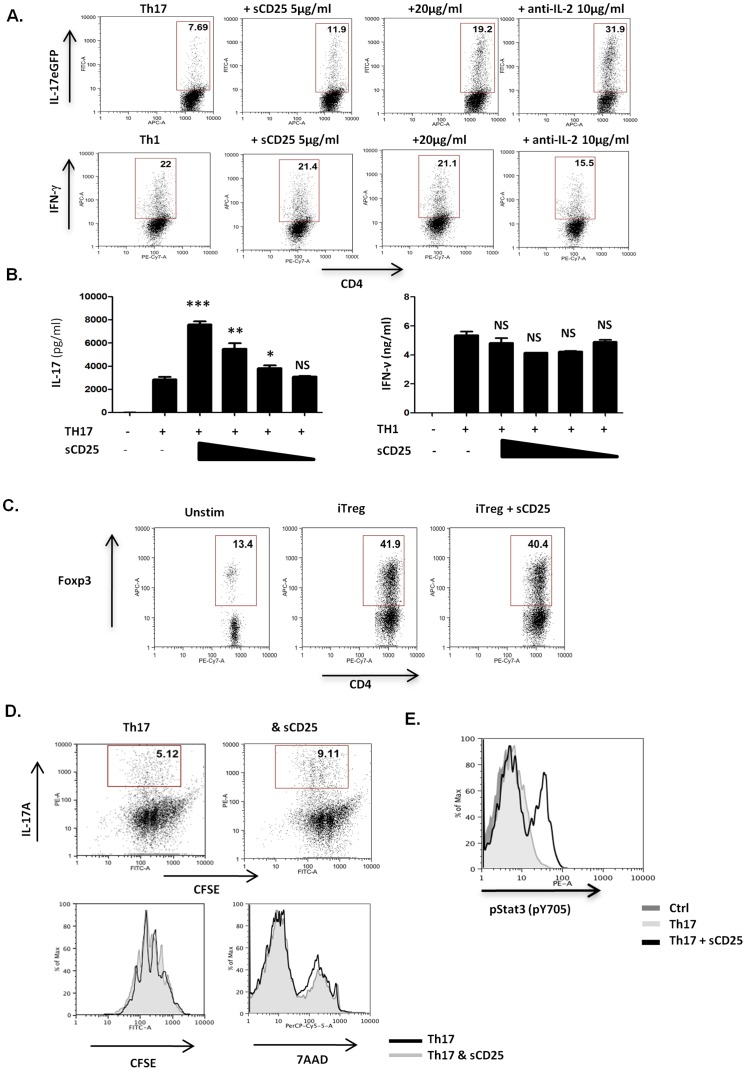
sCD25 enhances Th17 cell responses *in vitro*. (**A&B**) Purified naive CD4+ T cells were activated under either Th17 or Th1 inducing conditions (as described in methods) in the presence of a range of concentrations of sCD25 (20, 10, 5 or 1 µg/ml) or anti-IL-2 (10 µg/ml). Levels of IL-17A or IFNγ expression were determined after 96 hrs by (**A**) FACS and (**B**) ELISA. (**C**) Purified naive CD4+ T cells were activated under Treg inducing conditions, as described in methods, in the presence or absence of sCD25 (20µg/ml) and FoxP3 expression determined by FACS. (**D**) Naive CD4+ T cells were stained with CFSE (2.5 µM) prior to activation under Th17 conditions in presence or absence of sCD25 (20 µg/ml). After 96 hours, levels of intracellular IL-17A expression and CFSE dilution or 7AAD incorporation were determined by FACS. (**E**) Purified naive CD4+ T cells were activated under Th0, Th17 and Th17& sCD25 (20 µg/ml) conditions for 72 hours and levels of P-Stat3 (pY705) determined by FACS. All data are representative of 3 independent experiments. Statistical Significance determined by unpaired student's t-test, ∗ p≤0.05, **p≤0.01, ***p≤0.001.

sCD25 significantly enhanced the generation of Th17 type responses after 96 hours *in vitro* in a dose dependant manner and to a similar extent to an anti-IL-2 neutralizing antibody (Figure 3A). In contrast, no effects of sCD25 were observed on the generation of either Th1 or inducible Treg subsets (Figure 3 A, B & C). As Th17 cells are considered critical in driving the pathogenesis of EAE, these data are consistent with our earlier *in vivo* data (Figure 1). We also examined what influence sCD25 may have on Th17 cell proliferation and survival as IL-2 is a well established T cell growth factor and we observed an increase in the overall numbers of infiltrating CD4+ T cells in the spinal cords of treated mice (Figure 1C), Although sCD25 resulted in an increase in the proportion of cells expressing IL-17A, it did not affect either the rate of cell division as determined by levels of CFSE dilution, or levels of survival/cell death, as measured by incorporation of 7-AAD, under these conditions (Figure 3D). However, consistent with the enhanced generation of a Th17 response, we also observed increased levels of phosphorylation of STAT3 (Figure 3E). sCD25 also led to a marginal but reproducible increased expression of both IL-17F and the Th17 associated chemokine receptor CCR6, while levels of IL-22 expression were not affected (data not shown). These data clearly demonstrate that sCD25 can enhance Th17 cell development *in vitro* and suggest a mechanism through which sCD25 may increase autoimmune disease severity.

### sCD25 inhibits IL-2R signalling on Th17 cells through sequestering extracellular IL-2

**Figure 4:**
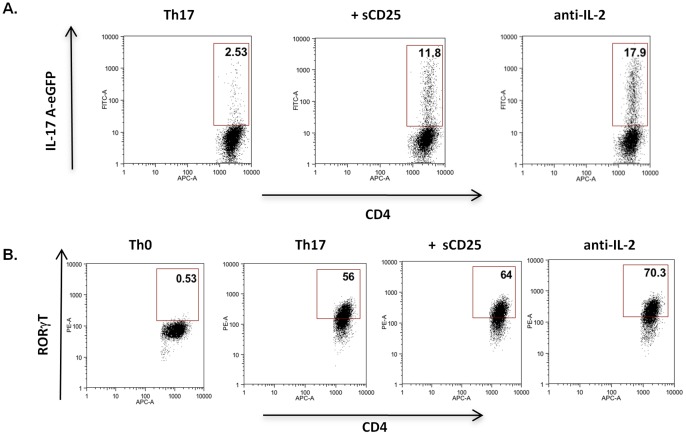
sCD25 acts early during Th17 development. Purified naive CD4+ T cells from IL-17AeGFP reporter mice activated under Th17 inducing conditions in the presence of sCD25 (20 µg/ml) or anti-IL-2 (10 µg/ml) for 48 hrs and examined for levels of (**A**) IL-17A and (**B**) RORγT expression by FACS. All data are representative of at least 3 independent experiments.

Recent reports have demonstrated that CD4+ FoxP3+ve Tregs can enhance the generation of Th17 type responses early during the developmental programme by limiting the bioavailability of IL-2 through constitutive expression of CD25 on their cell surface [18], [19]. Given these observations, we investigated whether sCD25 could act in a similar way. Strikingly, the presence of sCD25 led to a significant enhancement of IL-17A (11% vs 2%) expression early during Th17 cell differentiation (48 hours) indicating that sCD25 also mediated its effects early during Th17 development (Figure 4A). Consistent with this observation, sCD25 also led to increased expression of the Th17 instructive transcription factor RORγT (Figure 4B).

**Figure 5:**
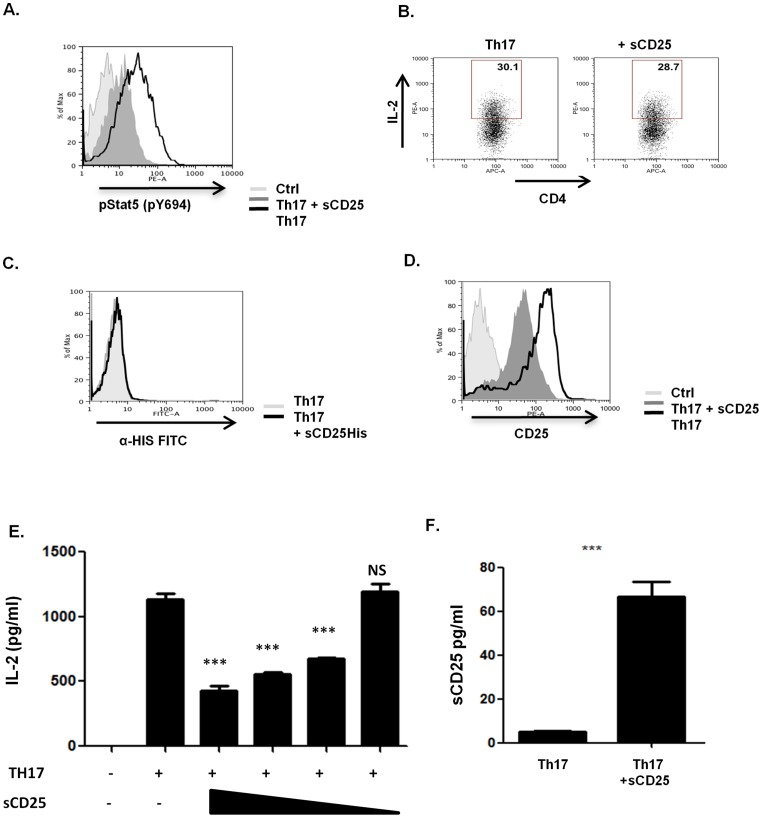
sCD25 inhibits IL-2R signalling by sequestering secreted IL-2. (**A–D**) Purified naive CD4+ T cells activated for 24 hrs under Th17 inducing conditions in the presence or absence of sCD25 and examined for levels of (**A**) p-Stat5 pY694, (**B**) intracellular IL-2 expression (**C**) surface binding of sCD25-His and (**D**) surface expression of endogenous CD25 by FACS analysis. (**E**) Levels of secreted IL-2 detected by ELISA 24 hours after Th17 activation in the presence of decreasing doses of sCD25 (20,10,5,1 µg/ml). (**F**) Levels of IL-2/sCD25 complex in the supernatants of Th17 cells activated for 24 hours in the presence or absence of sCD25 (20 µg/ml). Data shown as mean +/− standard deviation from triplicate experiments. All data are representative of at least 3 independent experiments. Statistical Significance determined by unpaired student's t-test, ***p≤0.001.

As the effects of sCD25 were similar to those observed for IL-2 neutralization [9], we examined whether signalling downstream of the IL-2R was affected in these cells. IL-2 is expressed early during the first 24 hours after TCR stimulation of CD4+ T cells and activation of Jak3-STAT5 dependent signal pathways in T cells during this time is considered to be largely driven by the autocrine effects IL-2. sCD25 significantly decreased levels of STAT5 activation in Th17 cells demonstrating its ability to inhibit signalling downstream of the IL-2R (Figure 5A). IL-2 dependent activation of STAT5 signalling is known to directly inhibit early programming events in the development of a Th17 response by blocking the induction of RORγT expression [9]. These data identify a novel mechanism whereby sCD25 enhanced the generation and development of proinflammatory Th17 responses through inhibiting the protolerogenic effects of IL-2R signalling.

To determine the precise mechanism through which sCD25 was mediating this inhibition we considered a number of possibilities. First, sCD25 may inhibit the levels of IL-2 expressed upon T cell activation (although IL-2 neutralization by monoclonal antibodies has previously been found to enhance IL-2 expression by inhibiting an auto-regulatory negative feedback loop [20]). We observed no differences between the levels of IL-2 expressed on a per cell basis either in the presence or absence of sCD25 after 24 hours (Figure 5B). Second, sCD25 may exert its effects at the cell surface by acting to either inhibit appropriate assembly of the heterotrimeric receptor complex or inhibit IL-2 binding. To examine this possibility we used a His-tag on the soluble form of the receptor to discriminate between soluble and surface expressed forms of CD25. However, we were not able to detect any binding of sCD25 to the cell surface during the first 24 hours after activation (Figure 5C). In contrast, the presence of sCD25 did significantly inhibit the upregulation of endogenous surface CD25 expression (Figure 5D). This observation further indicated a role for sCD25 in inhibiting IL-2R signalling as IL-2 is recognised as an important mediator in driving surface CD25 expression early during T cell activation. Third, we investigated the possibility that sCD25 may act to sequester secreted IL-2 in the T cell microenvironment. Significantly, sCD25 inhibited the detection of secreted IL-2 by ELISA in a dose dependent fashion suggesting its ability to sequester secreted IL-2 (Figure 5E). The specific interaction between sCD25 and IL-2 was also demonstrated using a mixed ELISA approach of capture with an anti-IL-2 antibody followed by detection with an anti-CD25 antibody. Using this approach, significant levels of the IL-2/sCD25 complex were detected in the supernatants of CD4+ T cells activated under Th17 conditions in the presence of sCD25 (Figure 5F).

Together these data demonstrate the immunomodulatory activity of the soluble form of the IL-2R alpha chain *in vivo* for the first time and indicate that these effects are mediated by its capacity to sequester secreted IL-2.

## Discussion

The expression of the heterotrimeric IL-2 receptor on the surface of T cells plays a pleiotropic role in directing T cell responses. One such critical non-redundant role is the maintenance of peripheral T cell tolerance. This occurs through its promotion of the induction and persistence of regulatory T cell subsets while also acting directly to inhibit the generation of Th17 type responses which are considered to be critical in driving autoimmune disease [1]. Such observations are thought to form the mechanistic basis for the close association between mutations at the *CD25* gene locus and enhanced susceptibility to a number of autoimmune diseases in humans.

Similar to other cytokine receptors expressed at the cell surface, the individual chains of the IL-2R are also known to exist in soluble form in serum [21], [22]. In particular, stable expression levels of soluble CD25 observed in healthy adults has underscored its clinical use as a biomarker for a variety of inflammatory conditions [23]. Unlike other soluble cytokine receptors, no evidence exists for an alternative splice form at the *CD25* gene locus which encodes a specific soluble CD25 protein. Consequently, the generation of the soluble form of this receptor is thought to occur through proteolytic cleavage at the cell surface by as yet unidentified proteases [24]. Levels of sCD25 generation increase upon T cell activation *in vitro* and enhanced levels observed *in vivo* are thought to be directly related to the magnitude T cell mediated inflammatory responses. However, recent analysis of specific autoimmune susceptibility alleles at the *CD25* gene locus has uncovered a direct association between increased disease susceptibility, disease severity and increased levels of sCD25 [10], [11]. These studies indicate that sCD25 may play an important mechanistic role in driving disease pathogenesis.

As expression of all three chains of the IL-2R signalling complex on the cell surface are known to be required for efficient IL-2 binding and the subsequent activation of downstream signalling events [25], whether sCD25 has any physiological relevance or is a mere by-product of T cell activation and expansion has remained controversial. Despite the lower affinity of CD25 for IL-2 when compared to the heterotrimeric IL-2R complex, sCD25 has been found to bind IL-2 efficiently and have immunomodulatory effects *in vitro* [10], [26]. It is also possible that sCD25 may interact with an as yet unidentified accessory protein(s) *in vivo* to enhance its affinity for IL-2. Along those lines, it is noteworthy that soluble IL-1RII is known to have its affinity for IL-1α/β enhanced almost 100 fold through its interaction with soluble IL-1R Accessory protein [27]. Although monomeric sCD25 has a molecular weight in the region of 40 kDa, it has previously been found to be present as part of a protein complex with a molecular weight in the region of 100 kDa in the synovial fluid of rheumatoid arthritis patients [28]. Although the accessory proteins involved in this complex were not identified, it was found to efficiently inhibit IL-2 mediated responses *in vitro*. Furthermore, sCD25 has been demonstrated to exist in homodimeric form, although whether this alters its relative affinity for IL-2 is unknown [29]. Studies are ongoing to determine whether sCD25 exerts its immunomodulatory effects in EAE through either oligomerization or binding accessory proteins *in vivo*.

Numerous studies have previously investigated the role of sCD25 in modulating T cell responses *in vitro*. These reports have often led to conflicting results with sCD25 having been variously described to both inhibit and enhance T cell responses. To our knowledge, no previous studies have examined the role of increased sCD25 in the clinical severity of an auto-immune disease. As sCD25 has been previously examined with respect to multiple sclerosis in humans, we chose a murine model of this disease to examine *in vivo* effects of sCD25. While a number of groups have demonstrated the capacity of sCD25 to inhibit IL-2 mediated proliferation of CD8+ cytotoxic T cell lines [28], [30], it is noteworthy that Maier et al. also demonstrated that sCD25 could inhibit IL-2 mediated STAT5 phosphorylation in primary CD4+ T cells while enhancing responses through the inhibition of activation induced cell death [10]. Our study further extends these *in vitro* findings and demonstrates that sCD25-mediated blockade of IL-2 signalling modulates T cell responses towards a Th17 phenotype.

Given the established role of IL-2 in mediating Treg homeostasis *in vivo* [3], it is surprising that we did not observe any effects on Treg subsets in the presence of sCD25 in this study. Although we did not specifically examine whether sCD25 affected the suppressive function of Tregs, levels of Foxp3 expression both *in vitro* and *in vivo* clearly indicate that sCD25 did not impact Treg survival or persistence. Similarly, previous reports have found that IL-2 can play a role in enhancing Th1 mediated responses but these were not affected by sCD25 at the concentrations used in this study [31] (Figure 2A, 3B). As Th17 responses are clearly elevated under these conditions, these data may reflect differing levels of sensitivity among pathogenic versus regulatory CD4+ T cell subsets towards the effects of IL-2 signalling. Alternatively, it is possible that Tregs, which express constitutively high levels of surface CD25 (and the heterotrimeric IL-2R) in comparison to Th17 cells, may be competitively less sensitive to sequestration of circulating IL-2 by sCD25. Studies are ongoing to determine how limiting doses of IL-2 may differentially impact CD4+ T cell responses and how sCD25 might influence these events.

It is clear from our *in vivo* studies that the ability of sCD25 to enhance Th17 responses on a per cell basis is only observed in the periphery and not at the site of inflammation where although percentages of both Th17 and Th1 cells are remarkably unaltered upon sCD25 treatment, both are present in significantly increased numbers (Figure 1). Although this may reflect the effects of sCD25 on T cell expansion, this seems unlikely given that we observed no such effects *in vitro* (Figure 3D). A further possible explanation for this is an increased plasticity or inter-conversion between both subsets at the site of inflammation. However, this is unlikely given that the IL-17A eGFP mouse used in these studies allows the identification of cells which also have a legacy of IL-17A expression. As such, increased plasticity would be evident as an increase in the percentage of GFP+ve cells expressing IFNγ which was not detected. More likely, these data indicate that enhanced antigen-specific Th17 cells in the periphery can facilitate the infiltration of both pathogenic Th1 and Th17 cells to the site of inflammation as has been previously reported [32].

In direct relevance to this study, the use of humanized anti-CD25 antibodies is showing considerable promise as a potential therapeutic for Multiple Sclerosis [33]. Although efficacy for this approach has been demonstrated in early clinical trials, the exact mechanism through which these antibodies inhibit disease remains obscure. It is noteworthy that these antibodies bind sCD25 and block its ability to sequester IL-2 [34]. As levels sCD25 are elevated among MS patients [10], blockade of its immunomodulatory effects with anti-CD25 could conceivably play an important part in the mechanism of action of Anti-CD25.

Together these data demonstrate the immunomodulatory activity of the soluble form of the IL-2R alpha chain *in vivo* for the first time and indicate that these effects are mediated by its capacity to act as a decoy receptor for secreted IL-2. Although biochemical studies indicate that CD25 in isolation has a significantly lower affinity for IL-2 when compared to the heterotrimeric IL-2R complex, it has been demonstrated to bind IL-2 efficiently and its ability to suppress IL-2 mediated responses *in vitro* has been extensively reported [10], [13], [26], [30]. The association between elevated levels of sCD25 found in the sera of autoimmune patients and the presence of specific susceptibility alleles at the *CD25* gene locus offer perhaps the clearest indication that sCD25 plays a role in autoimmune pathogenesis [10]. Although whether elevated levels of sCD25 are causally linked to the pathogenesis of human autoimmune disease remains to be determined, our studies demonstrate that sCD25 can act to enhance Th17 cell responses and provide a novel mechanism which may explain these observations.
